# Using the Three-Step Test Interview to understand how patients perceive the St. George’s Respiratory Questionnaire for COPD patients (SGRQ-C)

**DOI:** 10.1007/s11136-015-1192-3

**Published:** 2015-11-28

**Authors:** Muirne C. S. Paap, Lukas Lange, Job van der Palen, Christina Bode

**Affiliations:** Department of Research Methodology, Measurement, and Data-Analysis, Behavioural, Management and Social Sciences, University of Twente, P.O. Box 217, 7500 AE Enschede, The Netherlands; University of Twente, Enschede, The Netherlands; Medical School Twente, Medisch Spectrum Twente, Enschede, The Netherlands; Department of Psychology, Health and Technology, Behavioural, Management and Social Sciences, University of Twente, Enschede, The Netherlands

**Keywords:** HRQoL, COPD, Interview study, Qualitative research, Cognitive interview

## Abstract

**Purpose:**

The aim of this study was to assess the experiences of patients with chronic obstructive pulmonary disease (COPD) while they were completing the St. George’s Respiratory Questionnaire for COPD patients (SGRQ-C), using qualitative research methods.

**Methods:**

Twenty Dutch COPD patients were recruited through pulmonary physicians [13 women; mean age = 63.3 years (SD = 11.4)]. A trained interviewer applied the Three-Step Test Interview which allowed the interviewer to follow the thought process of the patient filling out the SGRQ-C. The official Dutch translation of the SGRQ-C was used.

**Results:**

Patients missed a recall period for the Symptoms subscale; were uncertain how to interpret specific words and phrases like “good days”, “games”, and “housework”; were confused by long-item stems that included a list of activities; and were frustrated by the dichotomous format used for the majority of SGRQ-C items (true/false).

**Conclusions:**

Overall, patients were satisfied with the SGRQ-C. Nevertheless, making minor adjustments could further increase its quality. This includes reintroducing a recall period in the first set of items such as used in the previous version and splitting up items consisting of multiple activities. Furthermore, we recommend using the same response format (4 or 5 response categories) for all items.

## Background

Short questionnaires (4–8 items) are becoming increasingly popular in health care and are often favoured over more traditional questionnaires (40–90 items). Also in the field of chronic obstructive pulmonary disease (COPD), many of the newest disease-specific health-related quality of life (HRQoL) questionnaires are rather short, for example the COPD Assessment Test (8 items) [1], the Chronic Respiratory Disease Questionnaire—Short Form (8 items), and the Clinical COPD Questionnaire (10 items). Their popularity in health care is not surprising; short scales are generally quick and easy to administer, reducing burden on patients and clinicians. However, item reduction does come at a price. Whether one is willing to pay this price may depend on key properties of the instrument as well as the application one has in mind. Of particular interest are whether the instrument is unidimensional or multidimensional, and whether it is to be used for research purposes (estimating relationships between variables on group level) or individual decision-making. It has been shown that the impact of shortening a scale does not necessarily have a big impact on criterion validity^1^ and expected value differences between two populations if the instrument is unidimensional and if the loss of reliability is corrected for [2]. This implies that a short scale measuring a unidimensional construct may be safely used to test hypotheses regarding group differences, provided that a suitable model is used. If the instrument is multifaceted (multidimensional) on the other hand, reducing the number of items is likely to come at the expense of content validity. Using item response theory (IRT) to select the “best” items (highest discrimination parameters) for the shortened scale has been suggested as a possible solution, but it is not a universal remedy; to the contrary, it has been shown that positive error correlations among items (which may occur if an instrument is not perfectly unidimensional) can result in upward-biased discrimination parameters [3]. The price we pay for shortening a scale becomes especially steep in the context of individual decision-making. Assigning a person to a treatment group based on the cut score of a short scale has been shown to result in as little as 50 % consistent classifications [4]. Moreover, a recent study by Kruyen et al. [5] showed that using short scales led to an increased risk of drawing incorrect conclusions regarding change in individual patients. This risk can be mitigated by using items of high psychometric quality. The authors recommended using at least 20 items if the goal is to detect change in a clinical setting.

In this light, the legacy instrument the St. George’s Respiratory Questionnaire (SGRQ; 50 items) [6] is currently still among the best stand-alone instruments to measure disease-specific HRQoL in COPD patients. Not surprisingly, it is often used to assess the convergent validity^2^ of newly developed tests. Since its introduction in 1991, the SGRQ has been subjected to many validation studies, mostly focused on psychometric properties. With the validation of the American translation of the SGRQ came an important modification; the reporting period of the symptom items was shortened from 1 year to 1 month (4 weeks) [7]. More recently, the SGRQ was shortened and improved based on psychometric analyses using a sample of COPD patients; this adjusted version was named SGRQ-C [8] (see Table 1). Eight items were removed from the original test due to poor psychometric properties. Furthermore, the response choices in the Symptoms subscale were modified. The specific reporting period was abandoned, because “…it has been problematic for some users” (see the Appendix of [8]). A recent study using three different psychometric techniques showed support for the shortening of the SGRQ; in this study, 19 items were removed due to poor psychometric performance while maintaining a high level of reliability [9].

**Table 1:** Items from the “St. George’s Respiratory Questionnaire for COPD patients: SGRQ-C”

Item no.	Item
	*Questions about how much chest trouble you have*
1	I cough
2	I bring up phlegm (sputum)
3	I have shortness of breath
4	I have attacks of wheezing
5	How many attacks of chest trouble did you have during the last year?
6	How often do you have good days (with little chest trouble)?
7	If you have a wheeze, is it worse in the morning?
8	How would you describe your chest condition?
	*Questions about what activities usually make you feel breathless.* *For each statement please select the box that applies to you these days:*
9	Getting washed or dressed
10	Walking around the home
11	Walking outside on the level
12	Walking up a flight of stairs
13	Walking up hills
	*Some more questions about your cough and breathlessness*
14	My cough hurts
15	My cough makes me tired
16	I am breathless when I talk
17	I am breathless when I bend over
18	My cough or breathing disturbs my sleep
19	I get exhausted easily
	*Questions about other effects that your chest trouble may have on you.* *For each statement please select the box that applies to you these days:*
20	My cough or breathing is embarrassing in public
21	My chest trouble is a nuisance to my family, friends or neighbours
22	I get afraid or panic when I cannot get my breath
23	I feel that I am not in control of my chest problem
24	I have become frail or an invalid because of my chest
25	Exercise is not safe for me
26	Everything seems too much of an effort
	*These are questions about how your activities might be affected by your breathing*
27	I take a long time to get washed or dressed
28	I cannot take a bath or shower, or I take a long time
29	I walk slower than other people, or I stop for rests
30	Jobs such as housework take a long time, or I have to stop for rests
31	If I walk up one flight of stairs, I have to go slowly or stop
32	If I hurry or walk fast, I have to stop or slow down
33	My breathing makes it difficult to do things such as walk up hills, carrying things up stairs, light gardening such as weeding, dance, play bowls or play golf
34	My breathing makes it difficult to do things such as carry heavy loads, dig the garden or shovel snow, jog or walk at 5 miles per hour, play tennis or swim
	*We would like to know how your chest trouble usually affects your daily life.* *For each statement please select the box that applies to you because of your breathing:*
35	I cannot play sports or games
36	I cannot go out for entertainment or recreation
37	I cannot go out of the house to do the shopping
38	I cannot do housework
39	I cannot move far from my bed or chair
40	*How does your chest trouble affect you?* *Please select ONE:*
	(a) It does not stop me doing anything I would like to do
	(b) It stops me doing one or two things I would like to do
	(c) It stops me doing most of the things I would like to do
	(d) It stops me doing everything I would like to do

Copyright (2005) by Jones [30]. Reprinted with permission

The few studies that report on feasibility of the SGRQ and SGRQ-C indicate that some items are difficult to complete. For example, the reporting periods of the SGRQ vary from “the last 4 weeks” for the Symptoms subscale to “usually” or “these days” for the other subscales. Patients might fail to notice these differences in reporting periods [10]. The recall period has been removed in the SGRQ-C. It has also been suggested that two items pertaining to symptoms do not apply to patients with COPD (and that this would explain why COPD patients often skipped these items) [10, 11]. One study showed that 5 % of the patients were not able to complete the whole SGRQ [12].

Although the SGRQ and SGRQ-C are still very popular and have been subjected to careful psychometric scrutiny, little has been reported about the way patients perceive, interpret, and respond to the items. In this study, we aim to fill this gap in the literature by using a cognitive interview to gain insight into how COPD patients perceive the SGRQ-C, i.e. the thought process they have when responding to the items.

## Methods

### Patients

Twenty COPD patients (13 women; 11 inpatients) were recruited in a pulmonary clinic in Enschede, the Netherlands. We wanted to explore a broad range of views on the items in the SGRQ-C and used purposive sampling to ensure that there was sufficient variability in disease severity, age, gender, and patient status (inpatient/outpatient). The age of the patients ranged from 45 to 84 years (*M* = 63.2, SD = 11.4). Face-to-face interviews (20–65 min) were conducted by one of two trained interviewers during November 2013 through January 2014. The inclusion criteria for participating in this study were as follows: a medical diagnosis of COPD; sufficient oral and written mastery of the Dutch language; being able to answer questions in a face-to-face interview; and being able to complete a questionnaire. Five interviews were conducted at the patients’ homes, while the other interviews were performed at the clinic. The ethical review board of the University of Twente approved the study. All patients gave informed consent. This study did not need the approval of the Medical Ethical Review Board, according to European regulations.

### The Three-Step Test Interview

**Fig. 1:**
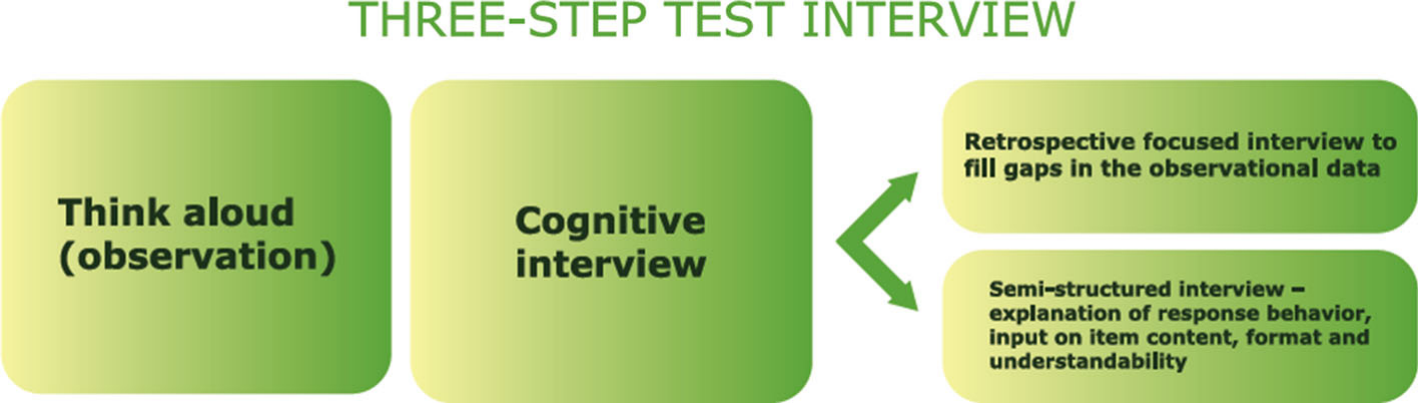
Three-Step Test Interview

The Three-Step Test Interview (TSTI) [13] combines observational and interviewing techniques to identify how items are interpreted, and whether problems occur during the completion of the questionnaire (see Fig. 1). The TSTI encompasses three consecutive steps: concurrent thinking aloud, a retrospective interview, and a semi-structured interview. As Hak et al. [14] describe, the TSTI is highly similar to the cognitive interview in that it uses think-aloud techniques and probing [15]. They suggest that a difference lies in the importance attributed to observing actual response behaviour real time: “The TSTI has been developed specifically as an instrument for discovering problems that occur during the completion of self-administered questionnaires by observing actual response behaviour” [14].

During the first step of the TSTI, the interviewer observes the respondent as they are completing the questionnaire and verbalising the thoughts they have while doing so. In this first step, the observer tries to avoid commenting or helping, but instead focuses on watching and listening attentively, and taking notes [16]. Respondents are encouraged to think aloud.

The second step consists of interviewing the respondent regarding their response behaviour, augmenting the data gathered in the first step. The interviewer can use spontaneous probes based on observations from step 1. The interviewer should only use probes that are relevant to the patient, and the interviewer should refrain from asking suggestive questions or putting words into the patient’s mouth [15].

During the third step, patients are invited to explain comments they made and the answering behaviour they showed during the earlier steps. In this step, patients can share their considerations and opinions about the questionnaire. The interpretation of the items and the thought process experienced by the patient when filling out the questionnaire is put in a broader (social-biographical) context [16]. Bode and Jansen argue that this allows the researcher to “…reach closer both to the response process and to the socio-biographical context of the respondent when compared with classical (cognition-based) forms of cognitive interviewing”. They conclude that “…this method [TSTI] seems more adequate than merely asking for interpretations of words and sentences in general”. See Hak et al. [14] and Bode and Jansen [16] for a comprehensive summary of the TSTI.

### The SGRQ-C

The SGRQ-C consists of 40 items (Table 1), of which 7 are scored on a Likert scale and 33 dichotomously. Both a total score and three subscale scores (Symptoms, 7 items; Activity, 13 items; Impact, 20 items) are usually calculated. The Symptoms subscale contains a recall period, which differs among versions (countries). For this study, the official Dutch translation of the SGRQ-C was used.

### Data analysis

The interviews were audio-recorded and transcribed verbatim. Analysis was performed by hand, on item level (except for comments made in step 3 which concerned the questionnaire as a whole), across the 3 steps of the interview. Comments and problems were labelled and subsequently grouped into categories. Coding was performed by LL, under supervision of CB and MP.

## Results

The data gathered in step 1 and 2 showed that every patient encountered problems with at least one of the items of the SGRQ-C; here, “problems” were defined as comments and/or missing values. The average amount of problems per patient was 7.9 (range 2–20). Item 7 (“If you have a wheeze, is it worse in the morning?”) was the only item in the questionnaire that did not cause any problems. Items 25 (“Exercise is not safe for me”), 35 (“I cannot play sports or games”), and 40 (“How does your chest trouble affect you?”) of the Impact subscale were the three items which created the most problems of all SGRQ-C items. Some patients misinterpreted the instructions and only ticked boxes if the item applied to them (for the dichotomous items). Only nine patients filled in every item. Some patients indicated that they missed items about certain important activities, such as riding a bicycle, hiking, how COPD influences job performance, and sex. Another frequently recurring remark was “these items are not applicable to my situation”, or “these items are only relevant to patients in the last GOLD stage”. All patients ticked the “false” box for item 39.

The data gathered in step 3 showed that 11 patients were satisfied with the questionnaire on the whole, in spite of what two of them called “minor issues”. Patients stated that the issues were highly applicable to them (1 patient), and easy to complete for the most part (2 patients). Four patients felt somewhat positive about the questionnaire, apart from the issues they had raised in the other two steps. Two patients were dissatisfied with the SGRQ-C, stating that the questions were unclear, strange, and hard to understand. One of these two patients stated that they had the feeling this questionnaire had clearly been designed by someone much cleverer than themselves. One patient refrained from commenting on the questionnaire itself, but underlined that they felt it was very important to conduct interviews to identify problems with questionnaires.

Eight categories of comments/problems were identified on item level: (1) problems with the response options; (2) difficult formulations/composite items; (3) patient suggests improvement; (4) questions containing problematic words; (5) patient does not like the item; (6) response would be dependent on weather; (7) instructions were misunderstood; (8) item is redundant/not applicable. These categories are discussed below. Categories 2 and 6 are addressed jointly. A summary of the findings is given in Table 2.

**Table 2:** Overview of the problems patients encountered while filling in the SGRQ-C

Item no.	Description	SGRQ-C scale	No. of patients indicating problems	Types of comments^a^
1	Cough	S	4	1
2	Phlegm	S	2	1
3	Short of breath	S	4	1
4	Wheezing	S	2	1, 6
5	Chest trouble	S	8	1, 2, 5
6	Good days	S	7	3, 4, 6
7	Wheeze morning	S	0	N/A
8	Chest condition	I	5	1, 4, 5
9	Breathless wash	A	2	7
10	Breathless walk 1	A	5	6, 7
11	Breathless walk 2	A	6	3, 4, 7
12	Breathless stairs	A	1	7
13	Breathless hills	A	4	4
14	Cough hurts	I	2	3, 7
15	Cough tired	I	1	7
16	Breathless talk	I	3	3, 7
17	Breathless bend	I	3	4, 5, 7
18	Sleep disturbed	I	2	3, 7
19	Exhausted	I	1	3
20	Embarrassing	I	3	1, 8
21	Nuisance family	I	5	1, 2
22	Panic	I	3	1, 3
23	Not in control	I	1	1
24	Invalid	I	4	3, 5
25	Not safe	I	9	3, 4
26	Effort	I	3	1, 2, 3
27	Slow wash	A	1	1
28	Slow bath	A	3	3
29	Slow walk	A	3	7, 8
30	Slow housework	A	5	1, 8
31	Slow stairs	A	1	8
32	Slow hurry	A	4	1, 3
33	Activities 1	A	6	1, 2, 3, 8
34	Activities 2	A	8	2, 3, 6, 8
35	Cannot sports	I	12	1, 2, 3, 4
36	Cannot recreate	I	3	4, 7
37	Cannot shop	I	3	3
38	Cannot housework	I	4	1, 4
39	Cannot move	I	5	3, 4
40	Doing things	–	11	1, 5, 7

^a^Eight categories of comments were identified: (1) problems with the response options; (2) difficult formulations/composite items; (3) patient suggests improvement; (4) questions containing problematic words; (5) patient does not like the item; (6) response would be dependent on weather; (7) instructions were misunderstood; (8) item is redundant/not applicable

### Problems with response options

Six patients were dissatisfied with the response options for one or more Symptoms items. Suggestions made by patients included adding the options “7 days a week” (items 1, 2, 3, 4), “sometimes” (items 1, 3), “mostly in the morning” (item 2), and “only with chest infections” (item 3). Two patients suggested item 5 should be an open question where patients could fill in a number; they felt the current response options did not reflect their situation well. Two patients suggested adding the option “sometimes” to one or more Activity items (27, 30, and 32), while four wanted to add “sometimes” to one or more Impact items (8, 20–23, 26, 33, 38, 40). One patient missed the option “only with chest infections” for item 8.

### Difficult formulations/composite items

Patients reported one or more formulation issues for items 5 and 26. Item 5 provoked a lot of negative evaluative comments from patients; one patient did not understand the question, while another said it was a “horrible question”. Three patients had an issue with the time span (the last year), indicating that it should be shortened to a month. Regarding item 26, one patient suggested replacing the word “seems” with the word “is”, while another patient commented that the formulation was unclear. Items 21, 33, and 34 were problematic because they consisted of two or more questions. As one patient stated about item 33: “Too many different things are included; they do not belong in one question”. Another patient stressed that some activities are harder than others: “Digging the garden you can still do. Jogging you cannot do, because you are not fit enough” (item 34).

### Patient suggests improvement

Patients offered a lot of suggestions for improving items. A theme that cut across items and subscales was the impact of the weather. Six patients indicated that the weather had a big impact on their symptoms (for example, the number of good days they have; item 6). For some items, patients missed a specified recall period (items 6, 18), distance (items 11, 39), or circumstances (items 14, 25, 37) and suggested adding this contextual information. For example, four patients indicated that the answer to certain questions depended on whether or not they had to do something independently or with the help of others/under supervision (exercising, doing grocery shopping). Patients had different solutions for the composite items; three respondents suggested dividing the activities and assigning each of them to a new item. Two patients suggested making different groupings of activities such as sport (1 item), housework (1 item), or heavy physical activities (1 item). One patient pointed out that the answers to certain questions depend more on comorbid disorders than their COPD (item 24), and suggested the formulation of question 24 should be adjusted to reflect this.

### Questions containing problematic words

Certain words or expressions were difficult for patients to interpret: “good days” (item 6; 3 patients), “hill” (item 13; 2 patients), “bend over” (item 17; 1 patient), “exercise” (item 25; 6 patients), “sports or games” (item 35; 9 patients), “recreation” (item 36; 2 patients), “housework” (item 38; 3 patients), and “far” (item 39; 2 patients). An often-heard remark was that these expressions were not specific/concrete enough.

### Patient does not like the item

There were different reasons for patients not to like a particular item. In some cases, patients stated these reasons, and in other cases, they did not (“what a horrible question”; item 5). Causes for frustration included answering categories not suiting the question (items 8, 40), patients being unsure what to answer because they avoid activities (item 17), and objection to certain expressions (“invalid”; item 24). Item 40 stands out: four patients were dissatisfied with the response categories because they were too much alike, and another three patients did not even fill in the item because they thought it was a “strange question”. All patients struggled with making the switch from dichotomous questions to a multiple-choice question at the very end and needed much longer time to complete this item compared to the other items.

### Instructions were misunderstood

A few patients misunderstood the written instructions. Although it clearly reads “For each statement please select the box that applies to you these days”, three patients only filled in dichotomous items if they were applicable to them. Another patient ticked both boxes for statements that were true for certain weather conditions but not for others (e.g. item 11). Some patients had trouble filling in a double negation correctly. For example, one patient mistakenly filled in “true” for item 36 (“I cannot go out for entertainment or recreation”), whereas she stated she was in fact still able to go out. Item 40 caused a lot of confusion; several patients ticked more than one box.

### Item is redundant/not applicable

Some items were reported to be too similar (items 29 and 32, 33 and 34). Patients were a bit unsure which box to tick if they did no longer perform a certain activity, or received help with the activity (e.g. items 30, 31).

## Discussion

The majority of patients had little difficulty filling in most of the SGRQ-C items. The issues that were raised are straightforward and could be readily addressed in an adapted version. Important issues include response format, the impact the weather has on symptoms, composite items, and specific formulations.

To our knowledge, this is the first study employing a cognitive interviewing technique to investigate how SGRQ(-C) items are interpreted by COPD patients since the SGRQ was designed. Using the TSTI allowed us to detect potential issues from the patient perspective. This approach has an important added value over psychometric analyses. For example, several authors proposed that items 5 and 7 are more suitable for evaluating difficulties with asthma rather than difficulties with COPD [10, 17]. Interestingly, the patients that were interviewed in this study reported no problems with item 7, and the comments on item 5 were not in line with the suggestions made in previous studies. Both items can be considered suitable for COPD patients. A recent study reported that the SGRQ could be shortened from 50 to 31 items without any substantial impact on the reliability [9]. Of the 19 “obsolete” items, seven were already removed during the development of the SGRQ-C [8]. Of the remaining 12, only two items proved highly problematic from the patient perspective (item 5 and 35).

Our findings indicate that the SGRQ-C is generally well received by COPD patients. Some improvements could be made to make it more user-friendly. We recommend the following:

1. Reintroducing a recall period of 1 month for the Symptoms items;
2. Using a fixed response format for all items;
3. Including information pertaining to (the influence of) weather conditions (or humidity) in item context or item instructions;
4. Avoiding composite items;
5. Providing clear instructions on how to complete an item if they no longer perform a certain activity or if they rely on the help of others for this.

Since the SGRQ-C items originate from the SGRQ, most of our recommendations are applicable to both the SGRQ-C and the SGRQ. The first recommendation is specific to the SGRQ-C, since a recall period of 1 month was in fact used in the SGRQ, but not in the SGRQ-C [8].

Ideally, patient input on acceptability, comprehensiveness, relevance, clarity, and ambiguity of items and instructions is sought during the development phase and in validating the questionnaire for use in a new population and/or language [18]. Several researchers have pointed out that making changes to an existing questionnaire can have important consequences; changing the wording of the question or response categories could change the meaning of the question and therefore the interpretation of the total score(s) [e.g. 19]. For that reason, many researchers shy away from improving existing questionnaires. We concur that ensuring comparability of scores is essential to measurement. However, it would be a mistake to simply assume that items are interpreted in the same way over time and by members of different populations, because that is not a matter of course. We recommend that reliability and validity be evaluated repeatedly for different types of applications and populations, using appropriate complementary methods (qualitative and quantitative).

So how do we maintain standardisation and comparability of scores, while allowing for the possibility that items may “behave” differently across different populations? The solution may lie in moving away from the static nature of traditional testing (“one size fits all”), and embracing a more flexible approach. If one is willing to trade in the sum score for an IRT [20]-based score, it becomes possible to compare the scores of two patients that answered a different set of items, as long as those items belong to the same domain and are calibrated on the same scale using an IRT model. Alternatively, one could use IRT to develop a so-called crosswalk between two instruments, that allows one to “translate” a score on one instrument into a score on another instrument (see [21, 22] for recent applications). This kind of approach allows for making improvements to an existing instrument, while maintaining comparability with scores which are calculated using the older version of the instrument.

Even if one decides not to make changes to an existing and much used legacy instrument, patient input on the quality of an instrument may prove useful for informing item adaptation when these items are to be included in an item bank on which a computerised adaptive test (CAT) is based [23, 24]. A CAT is a questionnaire that is tailored to the individual patient, while maintaining comparability across patients. Item selection in a CAT depends on the response given to previous items. In this way, the estimate of the outcome variable of interest (for example, HRQoL) is continuously adjusted, until a specific level of measurement precision (reliability) is reached. CATs are becoming increasingly popular in healthcare research, especially since the introduction of Patient-Reported Outcomes Measurement Information System (PROMIS). The PROMIS framework encompasses three major health domains which can be measured using a large number of IRT-calibrated item banks [25]. Many of these item banks include questions from legacy instruments, which were adapted after careful evaluation.

To verify whether our findings are truly attributable to the instrument itself, and not to the Dutch translation or culture, we discussed our findings with an expert; she is a native Dutch speaker who holds a university degree in English language and culture and has ample experience in English–Dutch and Dutch–English translation. She reviewed the original SGRQ-C and its Dutch translation in relation to our findings and reached the conclusion that only two comments were likely to be specific to Dutch culture. The word “hill” was found to be difficult to interpret for our patients; this may be due to the fact that the Netherlands is a predominantly flat country. Several patients indicated that they missed an item about “riding a bicycle”, which is a very common form of physical activity in the Netherlands and thus also may be a culture-specific finding.

In this study, we chose to let the concept of saturation guide choice of adequate sample size, as is common practice in qualitative studies. Saturation in this context means that no new problems in understanding and answering the SGRQ-C items arose. Although it is widely used, saturation remains a topic of debate, with respect to both the interpretation of the term and its utility. It was coined in the context of grounded theory research (thematic saturation: data collection is continued until new data are no longer generated), but has spread to other fields of qualitative research where it took on a new meaning (data saturation: fewer surprises, no new patterns in the data) [26, 27]. Several researchers have pointed out that stopping inclusion after saturation is reached in a pretest is no guarantee that all important problems have been identified. Blair and Conrad [28] and Perneger et al. [29] found strong positive relationships between sample size and the identification of problems. The lower the prevalence of a problem in the population, the larger the sample size requirements to detect it. Perneger et al. [29] reported that a problem with 10 % prevalence can be detected with a power of 80 % in a sample of 16 respondents, whereas 32 respondents are needed when the prevalence drops to 5 %. These findings spark an interesting debate: do we as researchers want to identify all possible problems, or are we interested in identifying particular types of problems? We concur with Blair et al. [28] that not all problems are of equal concern. We are interested in uncovering serious problems, problems that need to be addressed in order for respondents to be able to complete the items—even if the prevalence is low. With a sample size of 20 (as we had in this study), problems with a prevalence of 8 % and higher can be identified [29]. We cannot completely rule out that we missed certain problems, but by using purposive sampling, and a very thorough cognitive interviewing method, we feel confident that we maximised “the detectability of problems” and obtained a “high yield of problems” as Perneger et al. call it [29].

Overall, we felt that the TSTI did what it was designed to do: the think-aloud method and the cognitive interview allowed us to gain insight into how patients perceived and felt about the SGRQ-C items. The manual of the TSTI gives very clear instructions, making it an easy method to use for an interviewer with at least some interviewing experience. A potential drawback is that the TSTI is a very time-consuming method; two of the interviews lasted more than 1 h. Two patients explicitly stated that they were tired and that they lost focus as a result. For future research with the TSTI, we recommend dividing long questionnaires in two or more equal parts to reduce patient burden, especially if the patient is suffering from acute health problems as some of our patients were. We also recommend that interviewers take ample time to practise the think-aloud procedure with the patient before starting the actual interview, so that the patient will feel comfortable verbalising their thoughts.

The results of the current study could be used for a variety of purposes. They can be used by clinicians and researchers when selecting an appropriate HRQoL instrument to suit their needs and when interpreting SGRQ-C scores; the developers of the SGRQ and SGRQ-C could use them to improve the SGRQ and/or SGRQ-C further; and researchers developing an item bank to measure HRQoL in COPD patients could use them to select and improve items prior to inclusion. Taking the findings of our recent psychometric evaluation [9] and the current qualitative review of the SGRQ(-C) items together, we expect that the SGRQ-C would be an excellent starting point for a COPD-specific HRQoL item bank.

## Footnotes

1 Criterion validity in this context is measured by estimating the relationship between the scale of interest and a variable that serves as a criterion (e.g. outcome measure, legacy instrument, gold standard, expert rating).

2 Convergent validity in this context is typically measured by estimating the relationship between two scales that aim to measure the same or similar constructs.

## References

[1] Jones PW, Harding G, Berry P, Wiklund I, Chen WH, Kline Leidy N (2009). Development and first validation of the COPD Assessment Test. European Respiratory Journal.

[2] Heene M, Bollmann S, Bühner M (2014). Much ado about nothing, or much to do about something? Effects of scale shortening on criterion validity and mean differences. Journal of Individual Differences.

[3] Tuerlinckx F, De Boeck P (2001). The effect of ignoring item interactions on the estimated discrimination parameters in item response theory. Psychological Methods.

[4] Emons WH, Sijtsma K, Meijer RR (2007). On the consistency of individual classification using short scales. Psychological Methods.

[5] Kruyen PM, Emons WHM, Sijtsma K (2013). Assessing individual change using short tests and questionnaires. Applied Psychological Measurement.

[6] Jones PW, Quirk FH, Baveystock CM (1991). The St George’s Respiratory Questionnaire. Respiratory Medicine.

[7] Barr JT, Schumacher GE, Freeman S, LeMoine M, Bakst AW, Jones PW (2000). American translation, modification, and validation of the St. George’s Respiratory Questionnaire. Clinical Therapeutics.

[8] Meguro M, Barley EA, Spencer S, Jones PW (2007). Development and validation of an improved, COPD-specific version of the St. George Respiratory Questionnaire. Chest.

[9] Paap MCS, Brouwer D, Glas CAW, Monninkhof EM, Forstreuter B, Pieterse ME (2015). The St George’s Respiratory Questionnaire revisited: A psychometric evaluation. Quality of Life Research.

[10] Rutten-van Mölken M, Roos B, Van Noord JA (1999). An empirical comparison of the St George’s Respiratory Questionnaire (SGRQ) and the Chronic Respiratory Disease Questionnaire (CRQ) in a clinical trial setting. Thorax.

[11] Ferrer M, Alonso J, Prieto L, Plaza V, Monso E, Marrades R (1996). Validity and reliability of the St George’s Respiratory Questionnaire after adaptation to a different language and culture: The Spanish example. European Respiratory Journal.

[12] Ställberg B, Nokela M, Ehrs PO, Hjemdal P, Jonsson EW (2009). Validation of the clinical COPD Questionnaire (CCQ) in primary care. Health and Quality of Life Outcomes.

[13] Hak T, Van der Veer K, Jansen HAM (2008). The Three-Step Test-Interview (TSTI): An observation-based method for pretesting self-completion questionnaires. Survey Research Methods.

[14] Hak T, van der Veer K, Ommundsen R (2006). An application of the Three-Step Test-Interview (TSTI): A validation study of the Dutch and Norwegian versions of the ‘illegal aliens scale’. International Journal of Social Research Methodology.

[15] Willis GB (2005). Cognitive interviewing: A tool for improving questionnaire design.

[16] Bode C, Jansen H (2013). Examining the personal experience of aging scale with the Three Step Test Interview. Methodology.

[17] Karpinski N (2005). Validierung von Lebensqualitäts-Assessments bei chronisch–obstruktiven Atemwegserkrankungen [Validation of Quality of Life-Assessments in patients with Chronic Obstructive Pulmonary Disease].

[18] Fayers, P. M., & Machin, D. (2007).* Quality of life: The assessment, analysis and interpretation of patient-reported outcomes* (2nd ed.). Chichester: Wiley.

[19] Pool JJ, Hiralal SR, Ostelo RW, van der Veer K, de Vet HC (2010). Added value of qualitative studies in the development of health related patient reported outcomes such as the Pain Coping and Cognition List in patients with sub-acute neck pain. Manual Therapy.

[20] Embretson SE, Reise S (2000). Item response theory for psychologists.

[21] Ten Klooster PM, Voshaar MAO, Gandek B, Rose M, Bjorner JB, Taal E (2013). Development and evaluation of a crosswalk between the SF-36 physical functioning scale and Health Assessment Questionnaire disability index in rheumatoid arthritis. Health and Quality of Life Outcomes.

[22] Voshaar MAO, Ten Klooster PM, Taal E, Wolfe F, Vonkeman H, Glas CA (2014). Linking physical function outcomes in rheumatology: Performance of a crosswalk for converting Health Assessment Questionnaire scores to Short Form 36 physical functioning scale scores. Arthritis Care and Research (Hoboken).

[23] Van der Linden WJ, Glas CAW (2000). Computerized adaptive testing: Theory and practice.

[24] Paap MCS, Bode C, Lenferink LIM, Groen LC, Terwee CB, Ahmed S (2014). Identifying key domains of health-related quality of life for patients with chronic obstructive pulmonary disease: The patient perspective. Health and Quality of Life Outcomes.

[25] Cella DF, Riley W, Stone A, Rothrock N, Reeve B, Yount S (2010). The Patient-Reported Outcomes Measurement Information System (PROMIS) developed and tested its first wave of adult self-reported health outcome item banks: 2005–2008. Journal of Clinical Epidemiology.

[26] O’Reilly M, Parker N (2013). ‘Unsatisfactory Saturation’: A critical exploration of the notion of saturated sample sizes in qualitative research. Qualitative Research.

[27] Gaskell, G. (2000). Individual and group interviewing. In M. W. Bauer, & G. Gaskell (Eds.), *Qualitative researching with text, image and sound.* (pp. 39–57). London: SAGE Publications Ltd.

[28] Blair J, Conrad FG (2011). Sample size for cognitive interview pretesting. Public Opinion Quarterly.

[29] Perneger TV, Courvoisier DS, Hudelson PM, Gayet-Ageron A (2015). Sample size for pre-tests of questionnaires. Quality of Life Research.

[30] Jones PW (2005). St George’s Respiratory Questionnaire for COPD patients (SGRQ-C): Manual.

